# Morinda officinalis oligosaccharides alleviate depressive‐like behaviors in post‐stroke rats via suppressing NLRP3 inflammasome to inhibit hippocampal inflammation

**DOI:** 10.1111/cns.13732

**Published:** 2021-09-24

**Authors:** Zhifang Li, Hexiang Xu, Yi Xu, Guanfeng Lu, Qiwei Peng, Jiefang Chen, Rentang Bi, Jianzhuang Li, Shengcai Chen, Hongkai Li, Huijuan Jin, Bo Hu

**Affiliations:** ^1^ Department of Neurology Union Hospital Tongji Medical College Huazhong University of Science and Technology Wuhan China; ^2^ Institute of Science Beijing Tongrentang Co., Ltd. Beijing China

**Keywords:** hippocampus, Morinda officinalis oligosaccharides, NLRP3 inflammasome, post‐stroke depression

## Abstract

**Aims:**

Morinda officinalis oligosaccharides (MOOs), a traditional Chinese medicine, have been used to treat mild and moderate depressive episodes. In this study, we investigated whether MOOs can ameliorate depressive‐like behaviors in post‐stroke depression (PSD) rats and further explored its mechanism by suppressing microglial NLRP3 inflammasome activation to inhibit hippocampal inflammation.

**Methods:**

Behavioral tests were performed to evaluate the effect of MOOs on depressive‐like behaviors in PSD rats. The effects of MOOs on the expression of IL‐18, IL‐1β, and nucleotide‐binding domain leucine‐rich repeat (NLR) family pyrin domain containing 3 (NLRP3) inflammasome were measured in both PSD rats and lipopolysaccharide (LPS) and adenosine triphosphate (ATP) stimulated primary rat microglia by reverse transcription polymerase chain reaction (RT‐PCR), immunofluorescence and Western blot analysis. Adeno‐associated virus (AAV) was injected into the hippocampus to regulate NLRP3 inflammasome expression. The detailed molecular mechanism underlying the effects of MOOs was analyzed by Western blot and immunofluorescence.

**Results:**

MOOs can alleviate depressive‐like behaviors in PSD rats. PSD rats showed increased expression of IL‐18, IL‐1β, and NLRP3 inflammasome in the ischemic hippocampus, while MOOs reversed the elevation. NLRP3 downregulation ameliorated depressive‐like behaviors and hippocampal inflammation response in PSD rats, while NLRP3 upregulation inhibited the effect of MOOs on depressive‐like behaviors and hippocampal inflammation response in PSD rats. Moreover, we found that NLRP3 was mainly expressed on microglia. In vitro, MOOs effectively inhibited the expression of IL‐18, IL‐1β, and NLRP3 inflammasome in LPS + ATP treated primary rat microglia. We also showed that modulation of NLRP3 inflammasome by MOOs was associated with the IκB/NF‐κB p65 signaling pathway.

**Conclusion:**

Overall, our study reveals the antidepressive effect of MOOs on PSD rats through modulation of microglial NLRP3 inflammasome. We also provide a novel insight into hippocampal inflammation response in PSD pathology and put forward NLRP3 inflammasome as a potential therapeutic target for PSD.

## Graphical abstract

MOOs can ameliorate depressive‐like behaviors and hippocampal inflammation in PSD rat models via suppressing microglial NLRP3 inflammasome activation and IκB/NF‐κB p65 pathway.

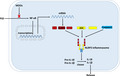

## 1 INTRODUCTION

Post‐stroke depression (PSD) refers to depression after cerebrovascular incident.^1^ The incidence of PSD is almost one‐third in stroke survivals.^2^ A large number of studies have indicated that PSD is positively associated with poor functional outcomes and increased mortality after stroke,^3^, ^4^ which gains public concerns. Currently, the treatment of PSD mainly relies on antidepressants such as selective serotonin reuptake inhibitors,^5^ while side effects remain a problem.^6^ Thus, it is urgent to find novel targets for therapeutic approaches to PSD.

In our previous study, we found that PSD rats showed suppressed glucose metabolism due to decreased glucose transporter‐3 expression in the neurons of the medial prefrontal cortex but not in the hippocampus, which is responsible for the occurrence of PSD.^7^ However, the hippocampus has been demonstrated to be an important regulator of emotion and cognition in the brain,^8^, ^9^ and evidence has also suggested the correlation between depression, hippocampal inflammation, and stroke. Studies have reported high serum levels of pro‐inflammatory biomarkers in depressive patients,^10^, ^11^ while several anti‐inflammatory agents such as celecoxib have been found to alleviate depressive symptoms in clinical trials.^12^ Inhibition of hippocampal inflammation has been proven to ameliorate depressive behaviors in mice.^13^ Moreover, cerebral ischemia can lead to a widespread inflammatory response in the brain, which can further affect the structure of the hippocampus.^14^ In clinical trials, stroke patients with higher serum IL‐18 or IL‐1β levels at admission have a higher risk of developing PSD.^15^, ^16^ Thus, excessive hippocampal inflammation may induce depression in stroke survivals.

Nucleotide‐binding domain leucine‐rich repeat family pyrin domain containing 3 (NLRP3) inflammasome, which is comprised of NLRP3, caspase‐1, and apoptosis‐associated speck‐like protein (ASC), plays an important role in inflammatory response via regulating the maturation of IL‐1β and IL‐18.^17^ In the central nervous system, NLRP3 inflammasome is mainly expressed on microglia^18^ and has been extensively reported to participate in brain disorders. In ischemic stroke, NLRP3 inflammasome activation can drive inflammation,^19^ increase BBB permeability^20^ and exacerbate recurrent strokes.^21^ In Alzheimer's disease and vascular dementia, NLRP3 inflammasome is activated^22^ and associated with cognitive impairment.^23^ Selective NLRP3 inflammasome inhibitor has been shown to alleviate neurological deficits and reduce IL‐1β production in intracerebral hemorrhage mice.^24^ Moreover, microglial NLRP3 inflammasome activation in the hippocampus has been found to mediate chronic stress‐induced depressive‐like behaviors in rats.^25^ These findings strongly imply that microglial NLRP3 inflammasome activation is firmly associated with depression. However, whether it is involved in PSD remains unknown.

Morinda officinalis oligosaccharides (MOOs), the traditional Chinese medicine extracted from *Morinda officinalis roots*, have long been used as a tonic to nourish the kidneys and strengthen immunity in humans.^26^ The Chinese Food and Drug Administration (CFDA) approved MOOs as a prescribed traditional herbal medicine for mild and moderate depressive episodes in 2012. There have been reports that oral administration of MOOs to depressed rodent animals can increase the monoamine and brain‐derived neurotrophic factor (BDNF) levels, which suggests the alleviation of depression.^26^, ^27^ In addition, studies have also reported that extracts from *Morinda officinalis* have an effect on colitis by regulating inflammation and cell apoptosis.^28^, ^29^

In this study, we found that MOOs could ameliorate depressive‐like behaviors and hippocampal inflammation in PSD rat models via suppressing microglial NLRP3 inflammasome activation. We further demonstrated the mechanism by which MOOs influence the IκB/NF‐κB p65 pathway to downregulate NLRP3 inflammasome expression in PSD rats. We also propose NLRP3 inflammasome as a potential therapeutic target for PSD and provide a novel insight into hippocampal microglial inflammation response in PSD pathology.

## 2 METHODS

### 2.1 Establishment of the PSD model

All animal experiments followed the Animal Research: Reporting In Vivo Experiments (ARRIVE) guidelines^30^ and were approved by the Animal Care and Use Committee, Huazhong University of Science and Technology. To produce the PSD model, transient middle cerebral artery occlusion (tMCAO) operation was performed on male Sprague‐Dawley rats (250–280 g) in accordance with previously described procedures.^7^ The filament was withdrawn for reperfusion after 2 h of tMCAO. Then, rats underwent individual housing and chronic unpredictable mild stress (CUMS) stimulation for up to 5 weeks after 7 days’ recovery from tMCAO surgery. The CUMS stimuli included water deprivation (12 h), food deprivation (24 h), 45° cage tilt, tail clamping (1 min), swimming in 4°C water (5 min), and overnight illumination (36 h). The sham groups only experienced the same surgical operation, but without tMACO, CUMS stimulation, or individual housing.

### 2.2 Drugs

MOOs were provided by Beijing Tongrentang Ltd. Co., Beijing, China and dissolved at a concentration of 10 mg/mL in distilled water. During the last 2 weeks of PSD model establishment, the PSD rats were orally administered with MOOs at a dose of 0.1 mg/g/d. The control groups were orally administered with vehicle of the same volume.

### 2.3 Behavioral tests

#### 2.3.1 Tail suspension test

Generally, adhesive tape was attached to the tail of the rats and they were then suspended 50 cm above the floor and observed for 6 min. The climbing time and immobility time were recorded as previously described.^31^

#### 2.3.2 Forced swimming test

On the first day, rats were placed in a transparent cylinder (50 cm, height; 30 cm, diameter) filled with water (30 cm, depth; 25°C, water temperature) for 6 min to adapt to the experiment environment. On the second day, rats were exposed to the same cylinder and observed for 6 min. The climbing time and immobility time were recorded as previously described.^32^

#### 2.3.3 Sucrose preference test

Two bottles containing 1% sucrose solution were placed in the rats’ cages 1 day before the test. On the second day, both bottles were withdrawn and the rats were kept away from water for 4 h before the test. To start the Sucrose preference test (SPT), one water bottle and one identical 1% sucrose solution bottle were placed in the rats’ cages after weighing both bottles. After 12 h, the locations of the two bottles were switched to avoid location preference. The SPT lasted for 24 h and both bottles were reweighed at the end of the test. Sucrose Preference (%) = Sucrose Volume/(Sucrose Volume + Water Volume) × 100%.

#### 2.3.4 Open field test

The Open field test (OFT) was performed to determine the motor ability of the rats before other behavioral tests to exclude motor deficiency as previously described.^33^ Briefly, rats were placed in a square wooden box (100 cm × 100 cm × 50 cm) for 5 min. Anilab software (Anilab Scientific Instruments Ltd. Co, Ningbo, China.) was used to analyze the traveling distance and average speed of the rats.

### 2.4 Microarray data

The microarray data of GSE78731 and GSE124387 were derived from Gene Expression Omnibus (GEO) databases (https://www.ncbi.nlm.nih.gov/geo/query/acc.cgi?acc=GSE78731, and https://www.ncbi.nlm.nih.gov/geo/query/acc.cgi?acc=GSE124387). The dataset GSE78731 included 5 wild‐type rats subjected to sham surgery and 5 ischemic rats subjected to MCAO, and its platform was based on the Agilent‐028279 SurePrint G3 Rat GE 8x60K Microarray. The dataset GSE124387 included 3 wild‐type rats and 3 depressed rats, and its platform was based on the HiSeq X Ten (Rattus norvegicus).

### 2.5 Differential expression analysis and functional enrichment analysis

R software and the limma package were used to identify the Differentially Expressed Genes (DEGs) of each GEO dataset. DAVID 6.8 (https://david.ncifcrf.gov/tools.jsp) was applied for functional enrichment analysis of DEGs and the top ten biological process terms were selected according to the *P*‐value of each term.

### 2.6 Viral injection

After 2 weeks of the tMCAO procedure, the rats were aseptically injected with 10% pentobarbital to induce anesthesia. Then, stereotaxic frames were applied to fix the anesthetized rats. Adeno‐associated virus (AAV) solution (1 × 10^13^ vg/ml) was injected into the hippocampus at a rate of 0.05 μL/min (total volume 2 μL). The coordinates of the four injection sites were as follows: (a) AP, −3.8 mm; ML, −1.5 mm; DV, −3.4 mm; (b) AP, −4.3 mm; ML, −3.0 mm; DV: −3.9 mm; (c) AP, −4.8 mm; ML, −5.2 mm; DV, −3.8 mm; (d) AP, −4.8 mm; ML, −5.2 mm; DV, −6.4 mm. AAV 2/8 serotype (pAKD‐CMV‐bGlobin‐eGFP‐H1‐shNLRP3, Obio Technology, Shanghai, China) was injected to downregulate NLRP3 expression and null vectors (pAKD‐CMV‐bGlobin‐eGFP‐H1‐shRNA‐NC, Obio Technology, Shanghai, China) were used for vehicle control. To overexpress NLRP3, recombinant AAV encoding NLRP3 (pAAV‐CMV‐Nlrp3‐3FLAG, Obio Technology, Shanghai, China) was injected, and AAV encoding null vectors (pAAV‐CMV‐MCS‐3FLAG, Obio Technology, Shanghai, China) were used for vehicle control. The behavioral tests were conducted 4 weeks after viral injection.

### 2.7 Primary microglial cell culture and treatment

Neonatal Sprague‐Dawley rats (P1‐P3) were used to isolate primary microglial cell cultures. Briefly, cerebral cortices were obtained and incubated in 0.25% trypsin/EDTA solution at 37°C for 15 min. Then, the digested cells were seeded into poly‐d‐lysine‐coated culture flasks in DMEM medium containing 10% FBS and 1% penicillin/streptomycin at 37°C in 5% CO2/95% air. The cultures were maintained for 10 days and replaced every 3 days. To separate microglia from the initial co‐culture, culture flasks were shaken at 250 rpm on a gyratory shaker for 1 h and the medium containing the detached microglia was collected and centrifuged. Then, purified microglia were re‐suspended and seeded in new culture flasks for further studies.

In the cell experiment, MOOs were dissolved at a proper concentration in DMEM. After pre‐treatment with MOOs (0, 1.25, 2.5, or 5 mg/mL) for 12 h, primary rat microglia were stimulated by LPS (Sigma, Louis, MO, USA) for 6 h and subsequent ATP (Sigma, Louis, MO, USA) for 30 min to induce inflammation.

### 2.8 Quantitative real‐time PCR

Total RNA was isolated using TRIzol reagent from Invitrogen. RNA extraction was reverse‐transcribed using the cDNA Synthesis Kit (Takara, Kyoto, Japan) according to the manufacturer's protocol, followed by the amplification of the cDNA using the SYBR Premix Ex Taq TM Kit (Takara, Kyoto, Japan). Then, quantitative real‐time PCR was performed using the thermocycler (Bio‐Rad, Hercules, CA, USA). The primers are shown below.

**Table:** 

Target	Sequence
RAT IL‐1β Forward	GCACAGTTCCCCAACTGGTA
RAT IL‐1β Reverse	TGTCCCGACCATTGCTGTTT
RAT IL‐6 Forward	AGGAGTGGCTAAGGACCAAGACC
RAT IL‐6 Reverse	TGCCGAGTAGACCTCATAGTGACC
RAT IL‐18 Forward	TGATATCGACCGAACAGCCAACG
RAT IL‐18 Reverse	GGTCACAGCCAGTCCTCTTACTTC
RAT TNF‐α Forward	GCATGATCCGAGATGTGGAACTGG
RAT TNF‐α Reverse	CGCCACGAGCAGGAATGAGAAG
RAT NLRP3 Forward	TGATGCATGCACGTCTAATCTC
RAT NLRP3 Reverse	CAAATCGAGATGCGGGAGAG
RAT ASC Forward	AGAGTCTGGAGCTGTGGCTACTG
RAT ASC Reverse	ATGAGTGCTTGCCTGTGTTGGTC
RAT Caspase1 Forward	GCCCAAGTTTGAAGGACAAA
RAT Caspase1 Reverse	GGTGTGGAAGAGCAGAAAGC
RAT Actin Forward	TGTCACCAACTGGGACGATA
RAT Actin Reverse	GGGGTGTTGAAGGTCTCAAA

### 2.9 Western blot

Total protein was obtained from the micro‐dissected hippocampus and primary rat microglia lysis. An equal amount of protein sample was separated by a 12% SDS‐PAGE gel and then transferred onto polyvinylidene fluoride membranes (Merck Millipore, Billerica, MA, USA). The following primary antibodies were used: anti‐NLRP3, 1:800, 12446, NOVUS; ASC, 1:300, 1‐78977, NOVUS; Caspase1, 1:700, A0964, AbClonal; IL‐1β, 1:700, A16288, AbClonal; IL‐18, 1:1000, 10663‐1‐AP, Proteintech; p65, 1:800, 10745‐1‐AP, Proteintech; p‐p65, 1:400, AP0475, AbClonal; IkBα, 1:1000, A1187, AbClonal; p‐IkBα, 1:500, AP0707, AbClonal; Actin, 1:1000, AC026, AbClonal; H3, 1:1000, AF0009, Beyotime. Membranes were incubated with primary antibodies overnight at 4°C, followed by secondary antibodies. β‐Actin and histone H3 were used as the housekeeping references for total and nuclear protein. Band intensity was measured with Image J software (NIH).

### 2.10 Immunofluorescence

Immunofluorescence was performed as previously described.^7^ The rats’ brains were fixed with 4% paraformaldehyde and then cut into 20‐mm sections. The following primary antibodies were used: anti‐NLRP3, 1:50, ab4207, Abcam; anti‐p65, 1:50, 10745‐1‐AP, Proteintech; anti‐Iba‐1, 1:200, ab5076, Abcam; anti‐GFAP, 1:50, A0237, AbClonal. Fluorescence‐labeled secondary antibodies were applied and 4′,6‐diamidino‐2‐phenylindole (DAPI, Invitrogen) was used to stain the nuclei. Samples were visualized using a TCS SP5 multiphoton laser scanning confocal microscope (Nikon, Tokyo, Japan).

### 2.11 Statistical analyses

All values were expressed as mean ± SEM. Shapiro‐Wilk test was used to evaluate the distribution of the data. Data that did not exhibit a Gaussian distribution was analyzed via a non‐parametric equivalent. For parametric analysis, differences between groups were evaluated by the one‐way ANOVA of Fisher's least significant difference test or the two‐tailed Student's t‐test using GraphPad Prism 7 software. Values of *p* < 0.05 were considered significant, and values of *p* < 0.01 were considered markedly significant.

## 3 RESULTS

### 3.1 MOOs alleviate depressive‐like behaviors in PSD rats

**FIGURE 1:**
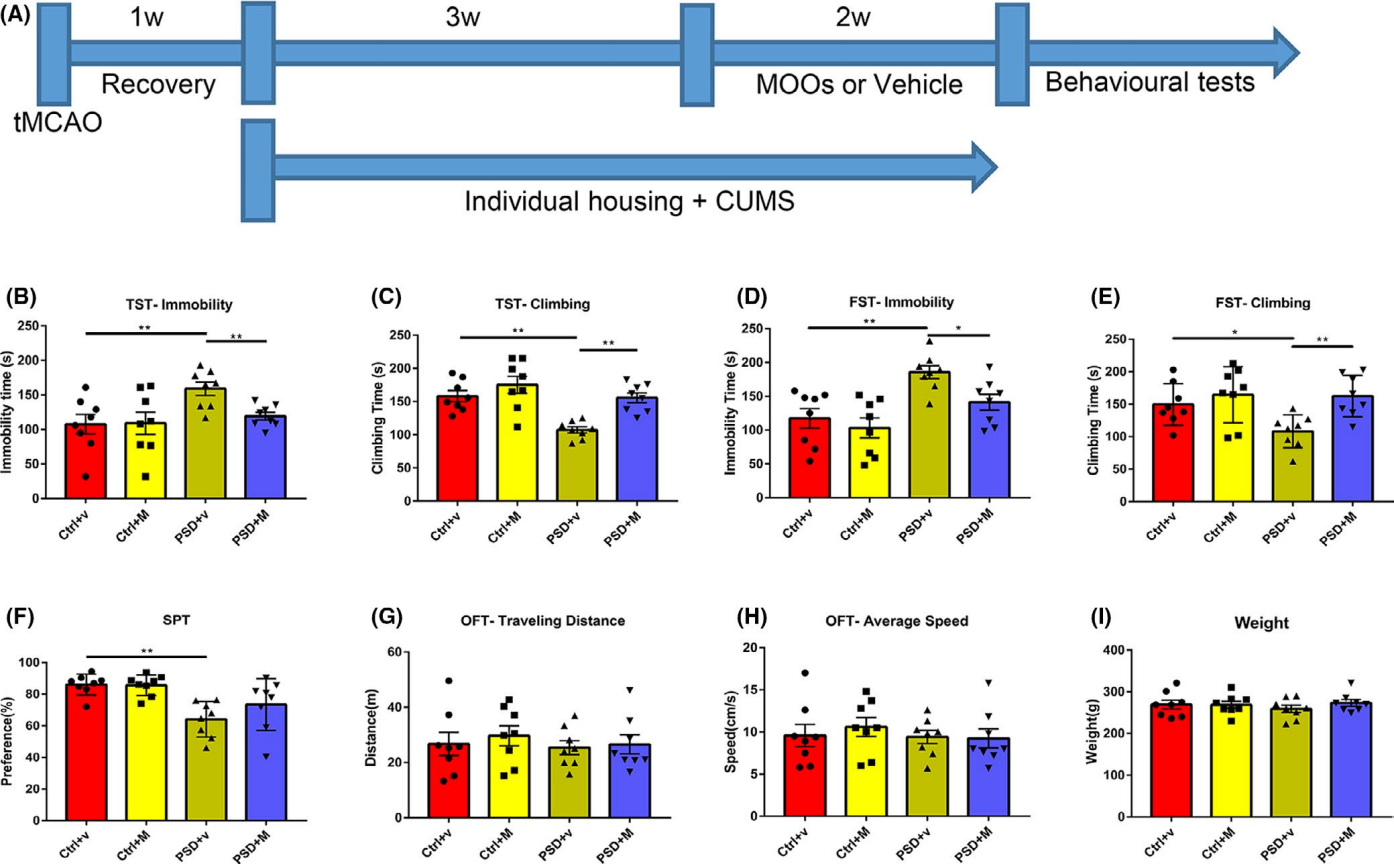
Morinda officinalis oligosaccharides (MOOs) alleviate depressive‐like behaviors in PSD rats. (A) An illustration of the experimental schedule. (B, C) The effect of MOOs on the immobility time (B) and the climbing time (C) in the TST test (*n* = 8). (D, E) The effect of MOOs on the immobility time (D) and the climbing time (E) in the FST test (*n* = 8). (F) The effect of MOOs on the sucrose preference in the SPT test (*n* = 8). (G, H) The traveling distance (G) and average speed (H) among these 4 groups in the OFT (*n* = 8). (I) The weight of four groups at the end of the experiment (*n* = 8). *p < 0.05, **p < 0.01. PSD, post‐stroke depression; tMCAO, transient middle cerebral artery occlusion; CUMS, chronic unpredictable mild stress; Ctrl +v, control group treated with vehicle; Ctrl +M, control group treated with MOOs; PSD +v, PSD group treated with vehicle; PSD +M, PSD group treated with MOOs; TST, tail suspension test; FST, forced swimming test; SPT, sucrose preference test; OFT, open field test

To study the antidepressive effect of MOOs, we used SD rats to establish the PSD model. We randomly divided the SD rats into 4 groups: sham groups administered with vehicle or MOOs (Ctrl + v group and Ctrl + M group) and PSD groups administered with vehicle or MOOs (PSD + v group and PSD + M group). As shown in Figure 1A, we first performed the tMACO operation on PSD rats. After 1 week's recovery from surgery, the PSD rats then underwent individual housing and CUMS stimulation for up to 5 weeks. We administered MOOs or vehicle to the PSD rats orally in the last two weeks of PSD model establishment. The sham groups only experienced the same surgical operation, without tMACO, CUMS stimulation or individual housing. Finally, we performed behavioral tests to assess their depressive‐like behaviors (Figure 1A).

From the behavioral tests, we found that depressive‐like behaviors were more obvious in PSD rats. The immobility time of PSD + v rats was dramatically increased, while the climbing time was significantly decreased in the Tail suspension test (TST) and Forced swimming test (FST) test compared with the Ctrl + v group (Figure 1B–E). In SPT test, PSD + v rats showed reduced sucrose preference (Figure 1F). Orally administration of MOOs to PSD rats can significantly ameliorate depressive‐like behaviors. In the TST and FST test, PSD + M rats showed more climbing time and less immobility time than PSD + v rats (Figure 1B–E), while the sucrose preference did not increase in PSD + M rats (Figure 1F), which means that MOOs treatment did not alleviate anhedonia in PSD rats. To exclude the possibility that motor dysfunction caused by stroke may have influenced the behavioral tests, we performed OFT and weighted the rats. No significant difference in traveling distance and average speed was found among the four groups in the OFT and rats’ weight in the four groups was the same (Figure 1G–I). From these results, it can be concluded that MOOs can alleviate depressive‐like behaviors in PSD rats.

### 3.2 MOOs inhibit the inflammatory response in the ischemic hippocampus of PSD rats

Cerebral ischemia can lead to the inflammatory response in the brain, and excessive inflammation, especially in the hippocampus, has been found to be associated with depression.^13^ Extracts of *Morinda officinalis* have also been found to regulate intestinal inflammation in mice.^28^ Thus, we speculated that MOOs might ameliorate depressive‐like behaviors via suppressing inflammatory response in the ischemic hippocampus of PSD rats.

**FIGURE 2:**
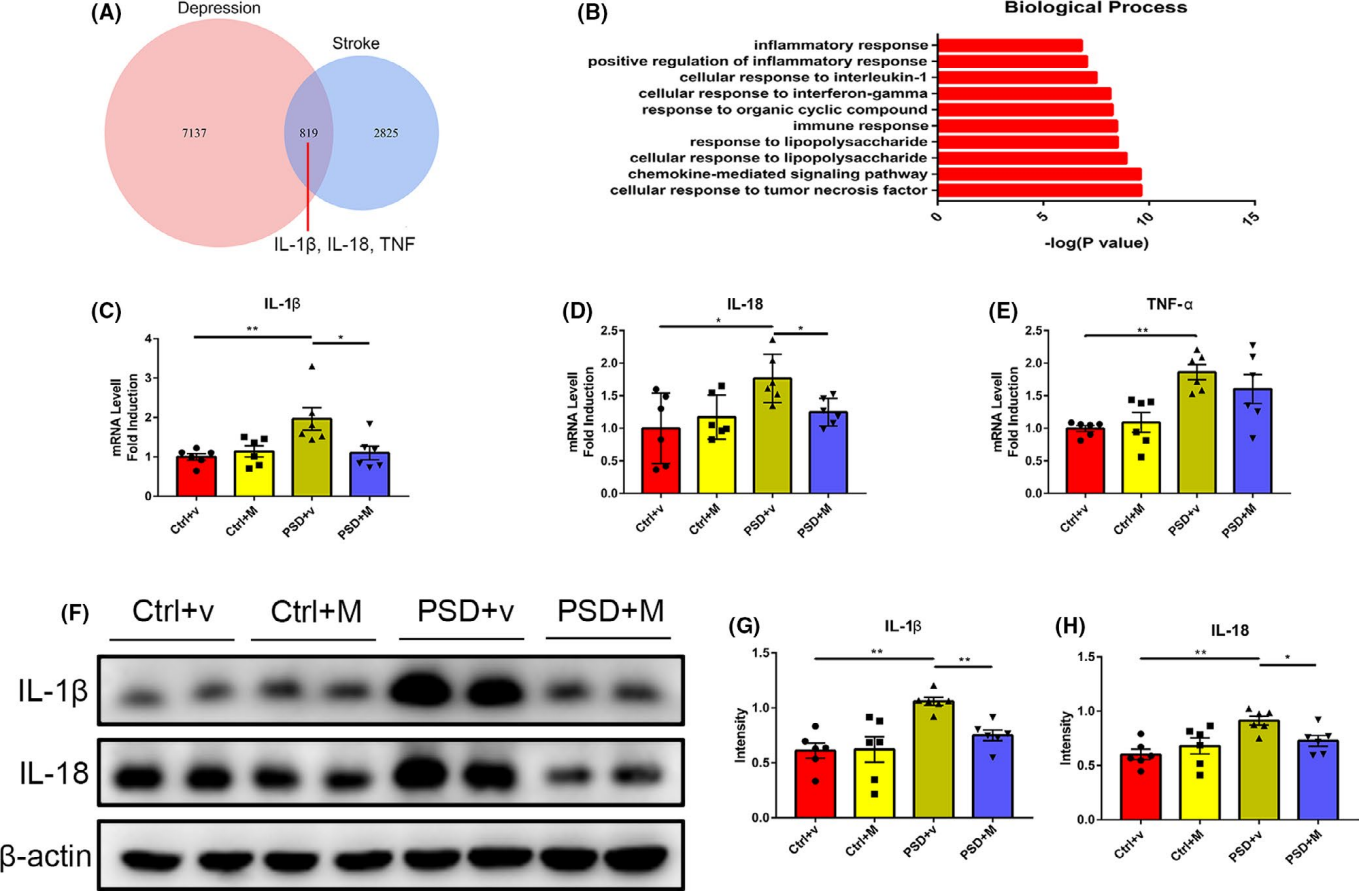
Morinda officinalis oligosaccharides (MOOs) inhibit the inflammatory response in the hippocampus of PSD rats. (A) Venn diagram comparing two lists of DEGs in the brain of rats with stroke or depression. (B) The top biological process GO terms of the overlapping DEGs. (C‐E) Quantification of the IL‐1β (C), IL‐18 (D), and TNF‐α (E) mRNA expression in the hippocampus of rats treated with vehicle or MOOs by RT‐PCR (*n* = 6). (F‐H) Western blot analysis showing IL‐1β (G) and IL‐18 (H) expression in the hippocampus of rats treated with vehicle or MOOs (*n* = 6). *p < 0.05, **p < 0.01. PSD, post‐stroke depression; DEGs, differentially expressed genes; Ctrl +v, control group treated with vehicle; Ctrl +M, control group treated with MOOs; PSD +v, PSD group treated with vehicle; PSD +M, PSD group treated with MOOs

We downloaded the stroke‐related gene expression profiling dataset GSE80681 and depression‐related gene expression profiling dataset GSE124387 from the GEO database and analyzed the DEGs using R software. Totally, 7956 DEGs were identified in GSE124387 and 3644 DEGs were identified in GSE80681. As shown in Figure 2A, 819 DEGs were detected in both GSE80681 and GSE124387. Using DAVID 6.8, we found that the overlapped 819 DEGs were enriched in several inflammation‐related biological processes (Figure 2B). The genes involved in the inflammation‐related biological processes are shown in Table 1. Among them, the levels of IL‐1β, IL‐18 as well as TNF‐α in serum have been reported to be elevated in PSD patients.^15^, ^16^ Next, we validated the mRNA expression of IL‐1β, IL‐18, and TNF‐α in the rat hippocampus using RT‐PCR analysis. As shown in Figure 2C–E, PSD + v rats showed increased mRNA expression of IL‐1β, IL‐18, and TNF‐α in the ischemic hippocampus compared with the control group. MOOs administration significantly reduced IL‐1β and IL‐18 mRNA expression (Figure 2C–D), while TNF‐α mRNA expression remained unchanged (Figure 2E). These results indicated that MOOs may affect IL‐1β and IL‐18 expression, rather than TNF‐α to perform an anti‐inflammation function. Then, we analyzed the protein expression of IL‐1β and IL‐18 in the ischemic hippocampus of PSD rats using Western blot analysis. Both IL‐1β and IL‐18 protein levels were significantly increased in the PSD + v rats compared with the control group, while MOOs administration reversed the change (Figure 2F–H). From these results, it can be concluded that MOOs treatment can suppress the inflammatory response in the ischemic hippocampus of PSD rats.

**TABLE 1:** Inflammation‐related terms in the top ten biological process terms according to the *p*‐value of each term

Terms	Counts	Genes
Cellular response to tumor necrosis factor	25	CD40, CALCA, CCL11, ADAMTS12, ICAM1, PYCARD, CCL9, CCL7, CCL6, CYP11A1, CCL4, CCL3, CCL2, CYP1B1, HAS2, CCL19, ADAMTS7, ENTPD1, ZFP334, **IL18**, CYBA, MMP9, COL1A1, FABP4, LCN2
Chemokine‐mediated signaling pathway	17	CCL11, GPR35, CXCR6, CXCL13, CXCL3, CXCL10, CCL9, CXCL11, CCL7, CCL6, CXCR3, CCL4, CCL3, CCL2, CCR6, CCL19, PF4
Cellular response to lipopolysaccharide	27	CD86, CSF3, CD40, CEBPE, LY96, CXCL3, **TNF**, CXCL16, ICAM1, PYCARD, KLRK1, THPO, PLAU, CYP11A1, MRC1, CCL2, STAP1, CD36, LBP, GBP2, ENTPD1, **IL18**, MMP9, CXCL10, **IL1β**, LCN2
Response to lipopolysaccharide	35	CD86, IL1RN, CD40, PTGER1, LY96, MGST2, ADM, CXCL13, CXCL3, **TNF**, LOXL1, C2, ICAM1, ADH4, THBD, EDNRA, THPO, CASP1, CCL2, LBP, JUNB, ENTPD1, SERPINA3N, IGF1, MMP9, CXCL10, CXCL11, SLPI, TH, **IL1β**, CFB, PF4, PTGES, FGF10
Cellular response to interleukin‐1	19	CD40, CCL11, PTGIS, SERPINA3N, ADAMTS12, MMP9, ICAM1, PYCARD, CCL9, CCL7, CCL6, CYP11A1, CCL4, LCN2, CCL3, CCL2, HAS2, CCL19, ADAMTS7
Positive regulation of inflammatory response	15	STAT5A, CCL11, ITGA2, **TNF**, IL17RB, CCL9, EDNRA, FABP4, CCL7, CCL6, CCL4, CCL3, CCL2, TLR10, TGM2
Inflammatory response	33	UCN, CD40, CALCA, PTGER1, CXCR6, CXCL13, CXCL3, **TNF**, KNG1, PYCARD, CCL9, CCL7, CCL6, CXCR3, CCL4, CCL3, SPP1, CCL2, CCL19, ITGB6, ANXA1, **IL18**, APOC3, CELA1, CYBA, SERPINA3N, CXCL10, CXCL11, **IL1β**, CRH, TLR10, TLR6, PF4

Tumor necrosis factor (TNF), Interleukin 18 (IL 18), Interleukin 1β (IL 1β).

### 3.3 MOOs negatively regulate the microglial NLRP3 inflammasome activation in the ischemic hippocampus of PSD rats

**FIGURE 3:**
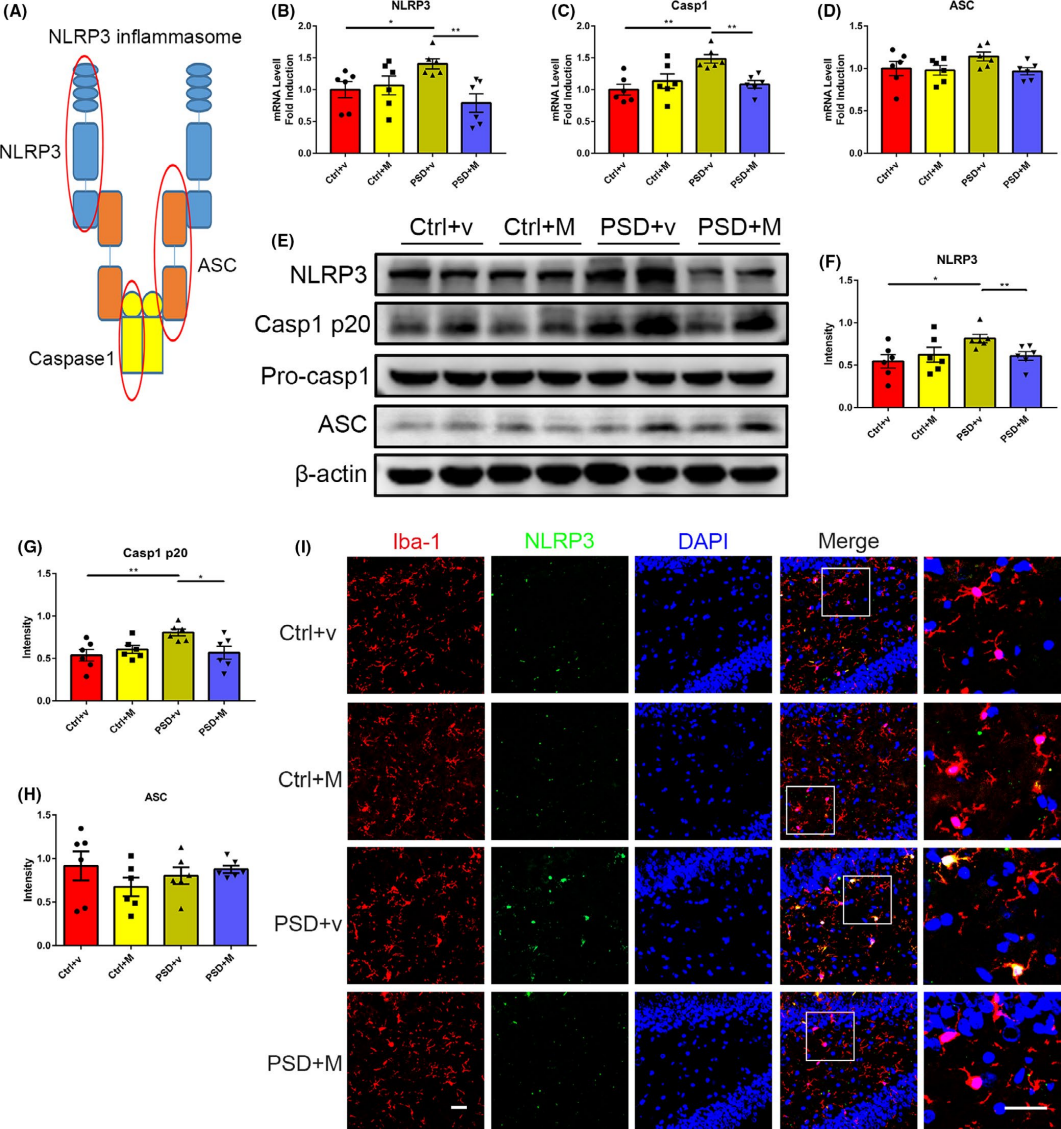
Morinda officinalis oligosaccharides (MOOs) suppress the microglial NLRP3 inflammasome activation in the hippocampus of PSD rats. (A) A presentative diagram of NLRP3 inflammasome. (B‐D) Quantification of the NLRP3 (B), Caspase1 (C), and ASC (D) mRNA expression in the hippocampus of rats treated with vehicle or MOOs by RT‐PCR (*n* = 6). (E‐H) Western blot analysis showing NLRP3 (F), Caspase1 p20 (G), and ASC (H) expression in hippocampus of rats treated with vehicle or MOOs (*n* = 6). (I) Immunofluorescence staining showing the expression of NLRP3 in the hippocampus (green). Microglia/macrophages were stained with Iba‐1 (red). Scale bar, 30 μm. *p < 0.05, **p < 0.01. NLRP3, nucleotide‐binding domain leucine‐rich repeat and pyrin domain containing receptor 3; Casp1, Caspase1; ASC, apoptosis‐associated speck‐like protein containing a caspase recruitment domain; PSD, post‐stroke depression; Ctrl +v, control group treated with vehicle; Ctrl +M, control group treated with MOOs; PSD +v, PSD group treated with vehicle; PSD +M, PSD group treated with MOOs; Iba‐1, ionized calcium binding adaptor molecule 1

NLRP3 inflammasome, which is comprised of NLRP3, Caspase1, and ASC (Figure 3A), has been reported to play a crucial role in inflammation by regulating the maturation and release of IL‐1β and IL‐18 in many diseases.^34^, ^35^, ^36^ In addition, several studies have revealed the association between depression and NLRP3 inflammasome activation.^25^ Thus, we proposed that MOOs may alleviate inflammation via suppressing NLRP3 inflammasome activation in the ischemic hippocampus of PSD rats. We measured mRNA and protein expression of NLRP3, Caspase1, and ASC in the hippocampus. PSD + v rats showed significantly increased NLRP3 and Caspase1 mRNA and protein expression, which was reduced in PSD + M rats (Figure 3B–H). This result indicated MOOs administration can abolish NLRP3 inflammasome activity in PSD rats. However, no differences in either ASC mRNA or protein expression were obtained among the four groups (Figure 3D and 3H). We further performed immunofluorescence staining and showed that NLRP3 inflammasome was mainly expressed in Iba‐1^+^ cells, the normally used marker for microglia (Figure 3I). PSD + v rats exhibited increased microglial NLRP3 inflammasome expression in the ischemic hippocampus, while MOOs can reverse the elevation (Figure 3I). In contrast, there was rare co‐localization of GFAP, a marker of astrocytes, and NLRP3 in the hippocampus in all four groups (Figure S1A). These results suggested that MOOs can suppress the microglial NLRP3 inflammasome activation in the hippocampus of PSD rats.

### 3.4 NLRP3 downregulation ameliorates depressive‐like behaviors and hippocampal inflammation response in PSD rats

**FIGURE 4:**
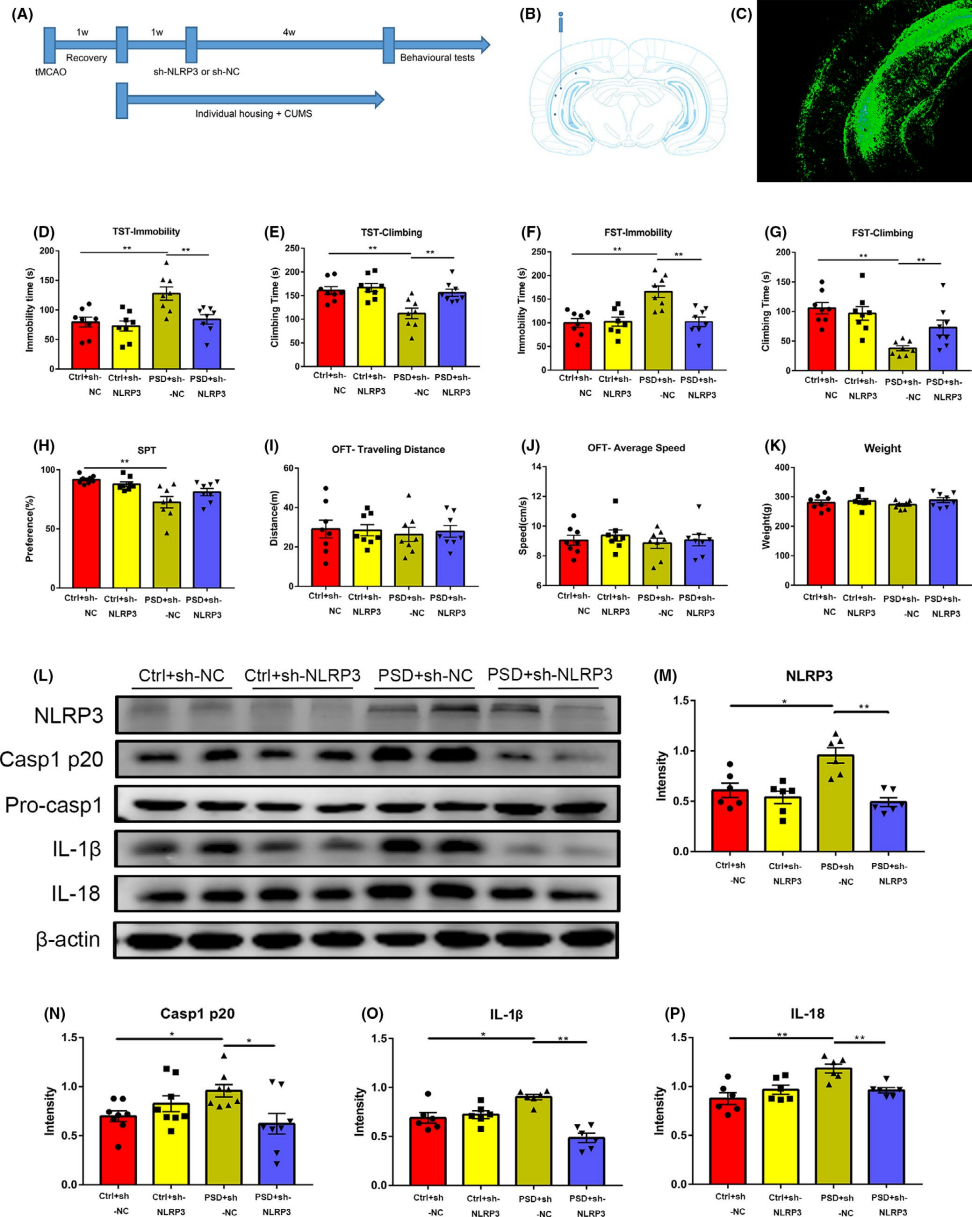
NLRP3 downregulation ameliorates depressive‐like behaviors and hippocampal inflammation response in PSD rats. (A) An illustration of the experimental schedule. (B) Illustration of the adeno‐associated virus (AAV) injection in rat brain. (C) Representative image shows the AAV‐infected hippocampal area in rat brain. (D, E) The effect of AAV‐induced NLRP3 downregulation on the immobility time (D) and the climbing time (E) in the TST test (*n* = 8). (F, G) The effect of AAV‐induced NLRP3 downregulation on the immobility time (F) and the climbing time (G) in the FST test (*n* = 8). (H) The effect of AAV‐induced NLRP3 downregulation on the sucrose preference in the SPT test (*n* = 8). (I, J) The traveling distance (I) and average speed (J) among these four groups in the OFT (*n* = 8). (K) The weight of 4 groups at the end of the experiment (*n* = 8). (L‐P) Western blot analysis of the expression of NLRP3 (M), Caspase1 p20 (N), IL‐1β (O), and IL‐18 (P) in the hippocampus of rats infected with sh‐NC or sh‐NLRP3 (*n* = 6). *p < 0.05, **p < 0.01. PSD, post‐stroke depression; tMCAO, transient middle cerebral artery occlusion; CUMS, chronic unpredictable mild stress; Ctrl + sh‐NC, control group with shRNA‐NC injection; Ctrl + sh‐NLRP3, control group with shRNA‐NLRP3 injection; PSD + sh‐NC, PSD group with shRNA‐NC injection; PSD + sh‐NLRP3, PSD group with shRNA‐NLRP3 injection; TST, tail suspension test; FST, forced swimming test; SPT, sucrose preference test; OFT, open field test

In order to further demonstrate the participation of NLRP3 inflammasome in the occurrence of PSD, we microinjected PSD rats with shRNA NLRP3 (PSD + sh‐NLRP3) or shRNA negative control (PSD + sh‐NC) using stereotaxic frames 2 weeks after tMCAO. Control groups were also injected with shRNA NLRP3 (Ctrl + sh‐NLRP3) or shRNA negative control (Ctrl + sh‐NC) (Figure 4A–C). After 4 weeks of injection, we found that the NLRP3 expression in the hippocampus of PSD rats was decreased (Figure 4L–M).

Then, we performed behavioral tests to evaluate the effect of NLRP3 downregulation on depressive‐like behaviors of PSD rats. Compared with PSD + sh‐NC rats, the immobility time was decreased and the climbing time was increased in PSD + sh‐NLRP3 rats in both TST and FST tests (Figure 4D‐G), while Ctrl + sh‐NLRP3 rats showed no changed behavior phenotype (Figure 4D‐G). Our data showed that NLRP3 downregulation can ameliorate depressive‐like behavior in PSD rats. In accordance to Figure 1F, no differences were detected between PSD + sh‐NC and PSD + sh‐NLRP3 on SPT (Figure 4H). Moreover, the traveling distance and average speed in the OFT and weight showed no difference (Figure 4I‐K), excluding the motor disability among the 4 groups.

To further investigate whether NLRP3 downregulation is adequate to alleviate hippocampal inflammation in PSD rats, we performed Western blot to measure the protein expression of pro‐Caspase1, Caspase1 p20, IL‐1β, and IL‐18 in the hippocampus. As shown in Figure 4L–P, shRNA NLRP3 transduction to PSD rats significantly decreased the Caspase1 p20, IL‐1β, and IL‐18 expression in the hippocampus compared with PSD + sh‐NC rats without affecting expression of pro‐Caspase1. Transduction of shRNA NLRP3 in control rats did not influence the Caspase1 p20, IL‐1β, and IL‐18 protein expression (Figure 4L–P). Taken together, these results confirmed that downregulating NLRP3 was adequate to abrogate hippocampal inflammation response in PSD rats.

### 3.5 NLRP3 upregulation abrogates the effect of MOOs on depressive‐like behaviors and hippocampal inflammation response in PSD rats

**FIGURE 5:**
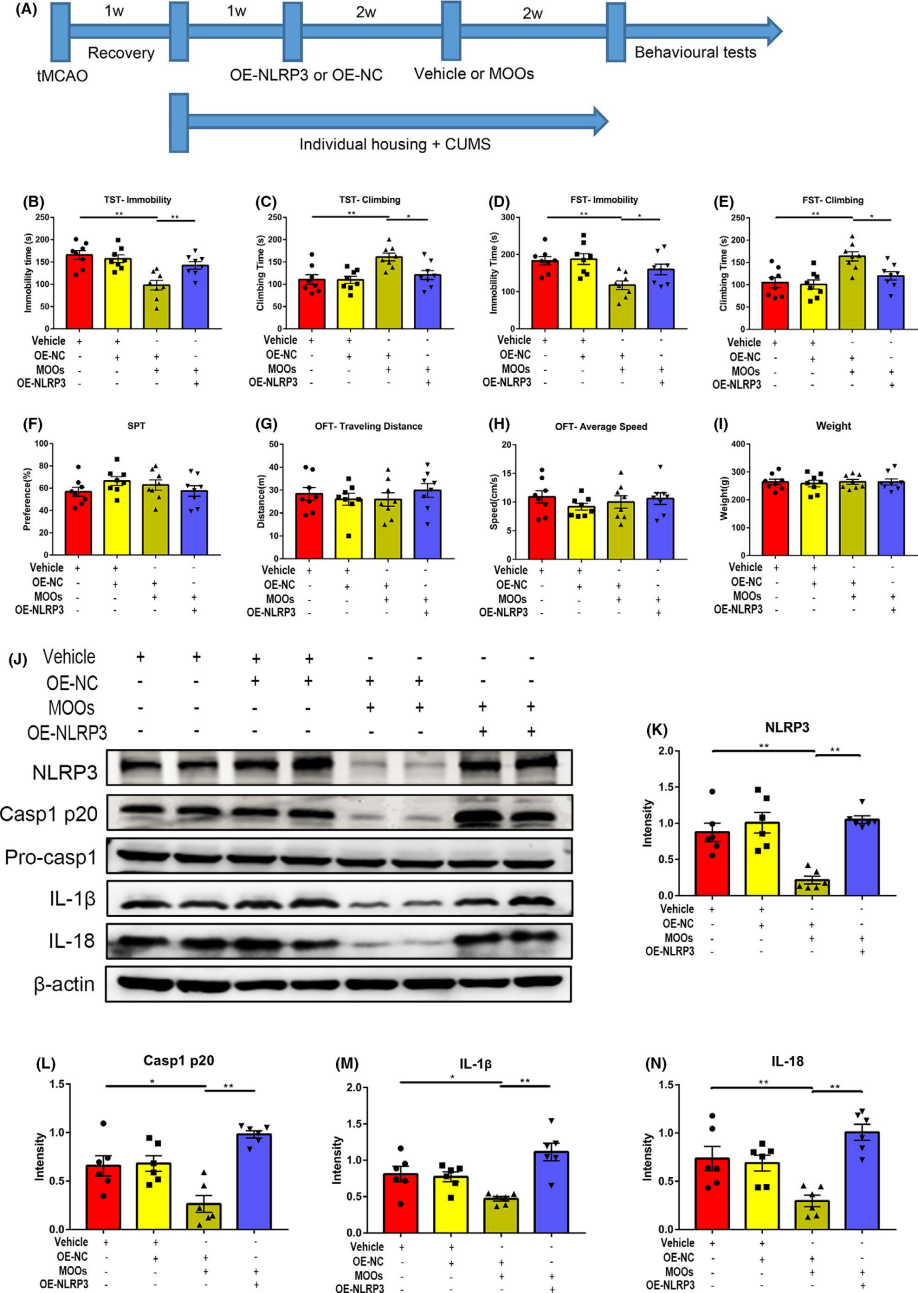
NLRP3 upregulation abrogates the effect of MOOs on depressive‐like behaviors and hippocampal inflammation response in PSD rats. (A) An illustration of the experimental schedule. (B, C) The effect of AAV‐induced NLRP3 upregulation on the immobility time (B) and the climbing time (C) in the TST test (*n* = 8). (D, E) The effect of AAV‐induced NLRP3 upregulation on the immobility time (D) and the climbing time (E) in the FST test (*n* = 8). (F) The effect of AAV‐induced NLRP3 upregulation on the sucrose preference in the SPT test (*n* = 8). (G, H) The traveling distance (G) and average speed (H) among these 4 groups in the OFT (*n* = 8). (I) The weight of 4 groups at the end of the experiment (*n* = 8). (J‐N) Western blot analysis of the expression of NLRP3 (K), Caspase1 p20 (L), IL‐1β (M), and IL‐18 (N) in the hippocampus of MOOs‐treated PSD rats infected with OE‐NC or OE‐NLRP3 (*n* = 6). *p < 0.05, **p < 0.01. PSD, post‐stroke depression; tMCAO, transient middle cerebral artery occlusion; CUMS, chronic unpredictable mild stress; OE‐NC, overexpression negative control; OE‐NLRP3, overexpression NLRP3; TST, tail suspension test; FST, forced swimming test; SPT, sucrose preference test; OFT, open field test

Next, to fully confirm whether MOOs function through NLRP3 inflammasome in PSD rats, we microinjected MOOs‐treated PSD rats with overexpression NLRP3 AAV (PSD +MOOs + OE‐NLRP3) or negative control AAV (PSD +MOOs + OE‐NC) using stereotaxic frames 2 weeks after tMCAO (Figure 5A). After overexpression NLRP3 AAV injection, we found that the NLRP3 expression in the hippocampus of the MOOs‐treated PSD rats was successfully increased (Figure 5J‐K).

Then, behavioral tests were performed to evaluate the effect of NLRP3 upregulation on MOOs‐treated PSD rats. PSD + MOOs + OE‐NLRP3 rats showed increased immobility time and decreased climbing time over PSD + MOOs + OE‐NC rats in both TST and FST tests (Figure 5B‐E). Similarly, PSD + MOOs + OE‐NLRP3 and PSD + MOOs + OE‐NC rats showed the difference in SPT, OFT, and weight (Figure 5F‐I). From these results, we can conclude that NLRP3 upregulation can abrogate the effect of MOOs on depressive‐like behaviors in PSD rats.

Hippocampal inflammation response was also tested in all 4 groups. As shown in Figure 5J‐N, overexpression NLRP3 AAV transduction to MOOs‐treated PSD rats significantly increased the Caspase1 p20, IL‐1β, and IL‐18 expression in the hippocampus compared with PSD + MOOs + OE‐NC rats. In conclusion, it is confirmed that NLRP3 upregulation could abrogate the effect of MOOs on inhibiting the hippocampal inflammation response in PSD rats.

### 3.6 MOOs inhibit the IκB/NF‐κB/NLRP3 signaling pathway in LPS+ATP treated primary rat microglia

**FIGURE 6:**
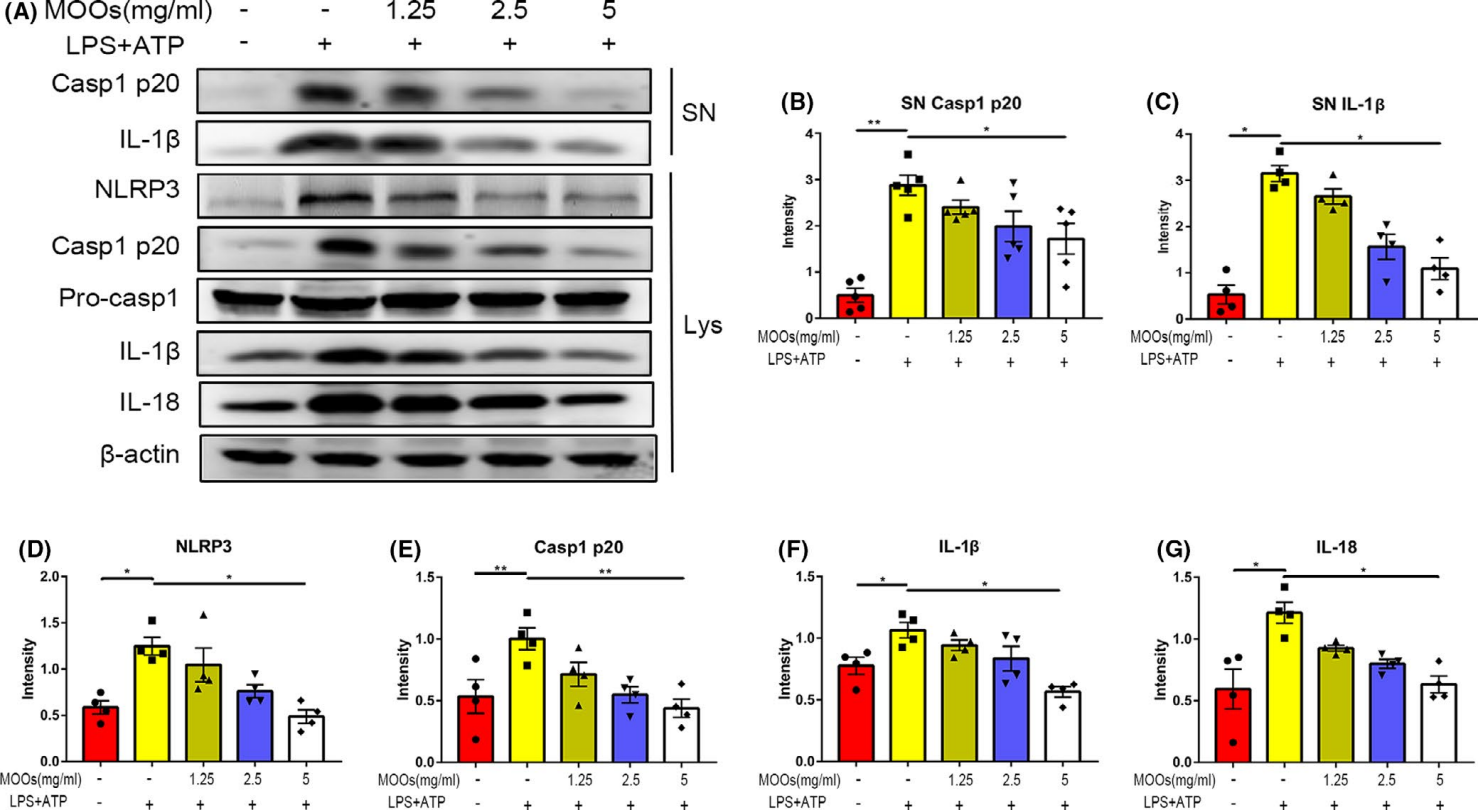
Morinda officinalis oligosaccharides (MOOs) inhibit LPS + ATP induced NLRP3 inflammasome activation in primary rat microglia. (A–G) Western blot analysis of the expression of Caspase1 p20 (B), IL‐1β (C) in supernatants and NLRP3 (D), Caspase1 p20 (E), IL‐1β (F), and IL‐18 (G) in primary rat microglia Lys after ATP + LPS and/or MOOs treatment (*n* = 4). *p < 0.05, **p < 0.01. SN, supernatants

Then, we tested the effect of MOOs on NLRP3 inflammasome activation in primary rat microglia in vitro. Primary rat microglia were treated with LPS for 6 h followed by ATP for 0.5 h to induce an inflammatory response. Meanwhile, MOOs were incubated at concentrations of 0, 1.25, 2.5, and 5 mg/mL in culture medium. As shown in Figure 6A–G, LPS + ATP stimuli significantly increased the expression of NLRP3, Caspase1 p20, IL‐1β, and IL‐18 in primary rat microglia lysate and caspase1 p20 and IL‐1β in the supernatant, which suggested LPS + ATP treatment‐induced NLRP3 inflammasome activation. MOOs administration significantly reduced NLRP3, Caspase1 p20, IL‐1β, and IL‐18 expression in cell lysate, as well as Caspase1 p20 and IL‐1β concentration in the supernatant in a dose‐dependent manner, without affecting the expression of pro‐Caspase1 (Figure 6A–G). These results indicated that MOOs can suppress LPS + ATP induced NLRP3 inflammasome activation in primary rat microglia.

**FIGURE 7:**
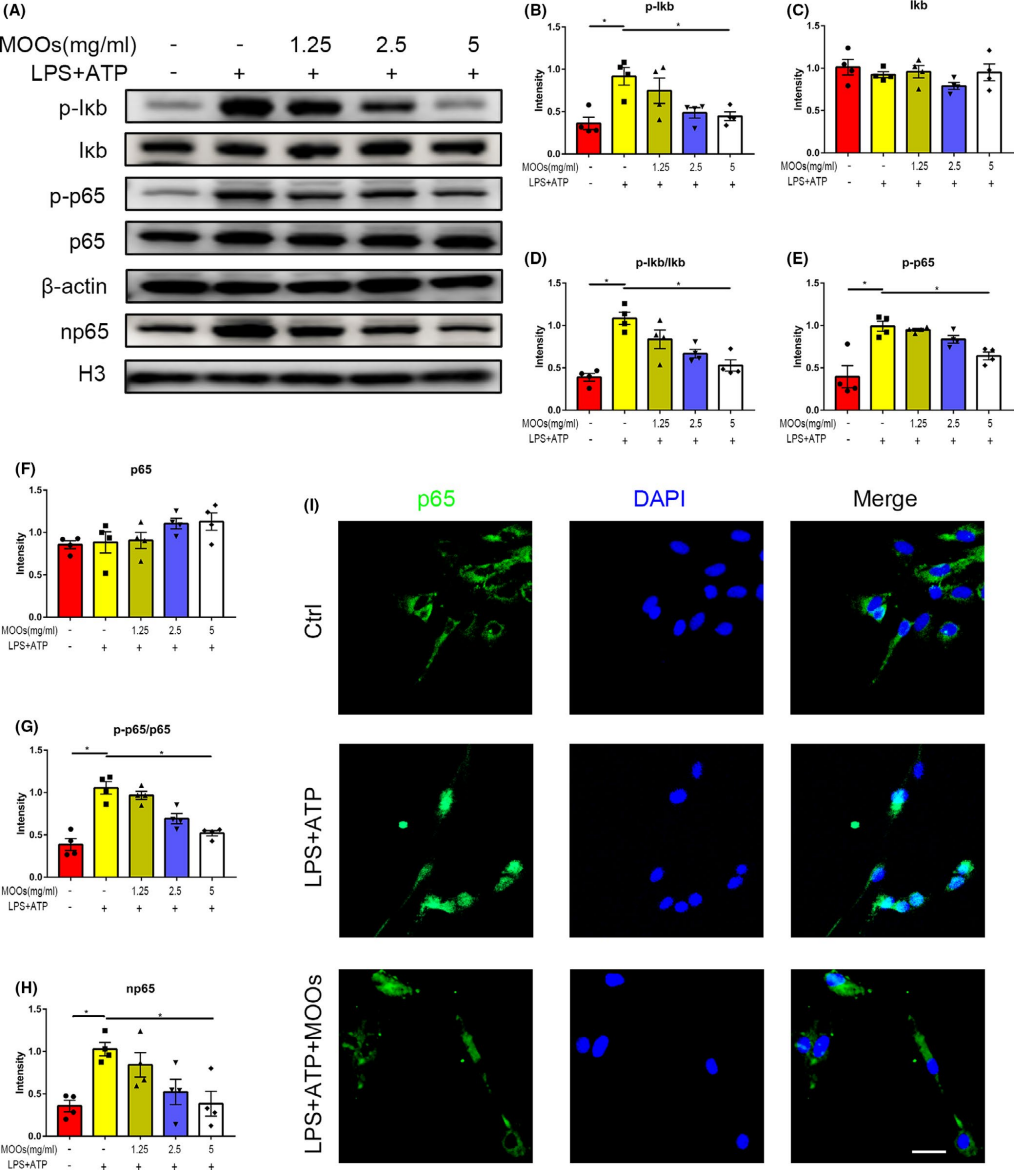
Morinda officinalis oligosaccharides (MOOs) suppress NF‐κB signaling pathway in LPS + ATP treated primary rat microglia. (A–H) Western blot analysis of the expression of p‐IκB (B), IκB (C), p‐p65 (E), p65 (F), and nuclear p65 (np65) (H) in primary rat microglia after ATP + LPS and/or MOOs treatment (*n* = 4). (I) Representative immunofluorescence images showing the translocation of p65 into nucleus. In the LPS + ATP + MOOs group, primary rat microglia were treated with MOOs at the concentration of 5 mg/mL. Scale bar, 20 μm. *p < 0.05, **p < 0.01

Previous studies have demonstrated that NLRP3 inflammasome activation can be regulated by transcriptional factor NF‐κB.^17^ Thus, we measured the pIκB, IκB, pNF‐κB p65, and NF‐κB p65 protein expression in LPS + ATP treated primary rat microglia in the presence or absence of MOOs. We found that levels of phosphorylated IκB (p‐IκB), p‐IκB/IκB, phosphorylated NF‐κB p65 (p‐p65) and p‐p65/p65 were significantly increased in LPS + ATP treated primary rat microglia, which indicated the activation of IκB/NF‐κB signaling pathway. MOOs treatment can reverse these changes, while total IκB and NF‐κB p65 levels were not influenced (Figure 7A–G). The activated NF‐κB p65 can translocate into the nucleus initiating the transcription. Accordingly, we found that nucleus p65 (np65) level was increased after LPS + ATP treatment, while MOOs significantly abrogated the effect (Figure 7A, H). Confocal microscopy also showed that MOOs treatment can lessen the nuclear translocation of NF‐κB p65 in LPS + ATP treated primary rat microglia (Figure 7I). In conclusion, MOOs may inhibit the IκB/NF‐κB signaling pathway to negatively regulate NLRP3 inflammasome in primary rat microglia.

### 3.7 MOOs suppress the IκB/NF‐κB signaling pathway in PSD rats

**FIGURE 8:**
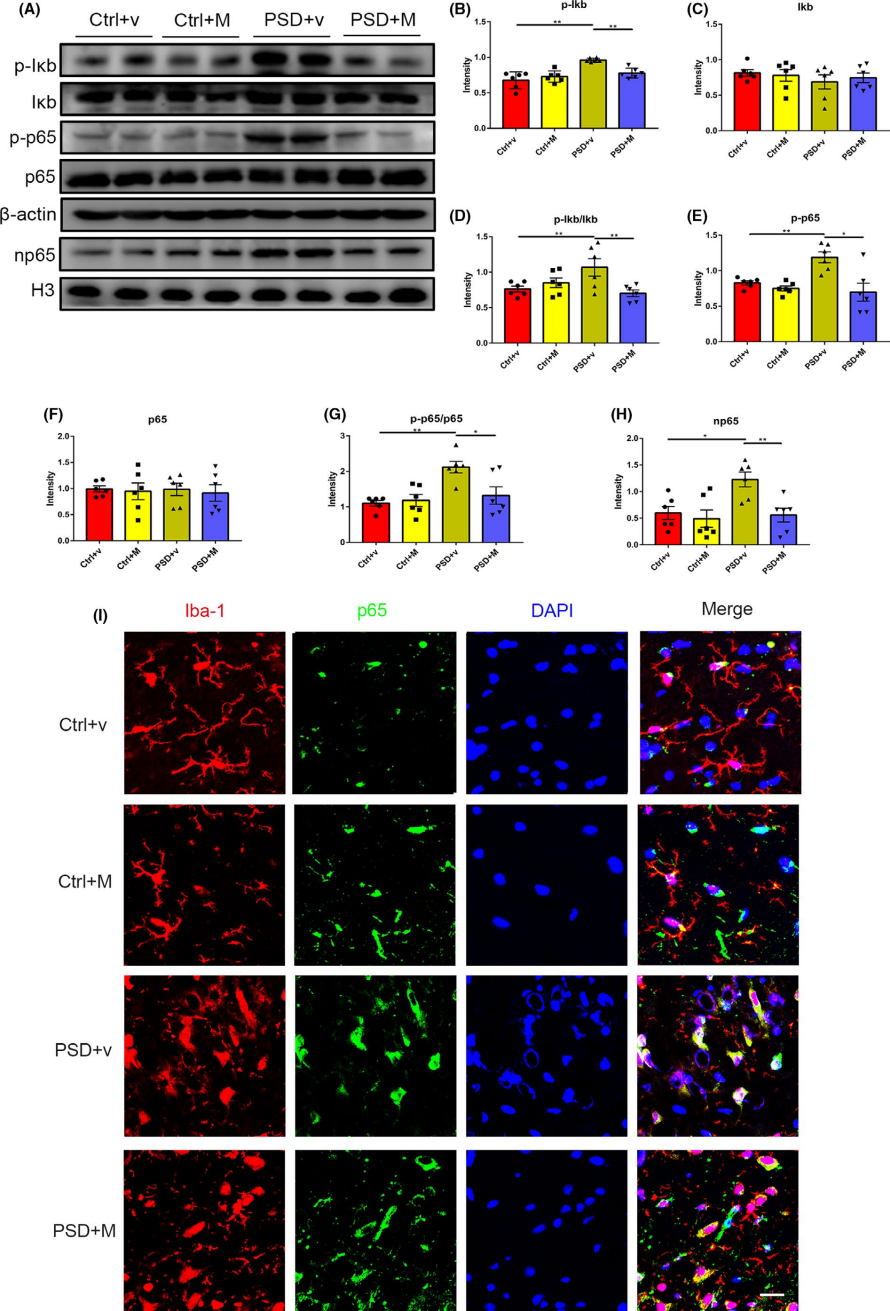
Morinda officinalis oligosaccharides (MOOs) suppress NF‐κB signaling pathway in PSD rats. (A–H) Western blot analysis of the expression of p‐IκB (B), IκB (C), p‐p65 (E), p65 (F), and nuclear p65 (np65) (H) in the hippocampus of rats treated with vehicle or MOOs (*n* = 6). (I) Representative immunofluorescence images showing the translocation of p65 into nucleus in microglia. Scale bar, 15 μm. *p < 0.05, **p < 0.01

To further confirm the effect of MOOs on the tIκB/NF‐κB signaling pathway in *vivo*, we measured the protein expression of p‐IκB, IκB, p‐p65, p65, and np65 in the ischemic hippocampus of PSD rats. We found that compared with the control group, the levels of p‐IκB, p‐IκB/IκB, p‐p65, p‐p65/p65, and np65 were increased in PSD + v rats. MOOs can reverse the elevation without affecting total IκB and p65 levels (Figure 8A–H). Further confocal microscopy was also performed to assess the nuclear translocation of p65 in microglia in PSD rats. We found that PSD +v rats showed higher nuclear translocation of p65 in microglia than Ctrl +v rats and MOOs can reduce this translocation (Figure 8I). Taken together, MOOs may inhibit NLRP3 inflammasome activation of PSD rats by suppressing the IκB/NF‐κB signaling pathway.

## 4 DISCUSSION

In this study, we demonstrated the antidepressive effect of MOOs, the herbal extracts from *Morinda officinalis* roots, in PSD rat models. We also revealed the mechanism by which MOOs alleviate hippocampal inflammation response by inhibiting microglial NLRP3 inflammasome activation. We further identified that MOOs may suppress the IκB/NF‐κB p65 pathway to downregulate NLRP3 inflammasome expression. The results indicated that NLRP3 inflammasome is a potential target for PSD treatment and highlighted the involvement of NLRP3 inflammasome activation in PSD pathology.

*Morinda officinalis* is one of the best‐known Chinese herbs and widely used as a Yang‐tonic agent to treat a wide range of diseases, such as osteoporosis, rheumatoid arthritis, impotence, dermatitis, Alzheimer's disease, and depression.^26^ Phytochemistry studies have revealed that *morinda officinalis* is comprised of variable chemical constituents, mainly including iridoid glycosides, anthraquinones, polysaccharides, oligosaccharides, organic acids, and volatile oils.^26^ MOOs are the inulin‐type fructooligosaccharides extracted from *morinda officinalis*, with 3–9 degrees of polymerization (DPs). Among them, DP5 has the largest proportion of MOOs.^37^ Previous studies have reported that oral administration of MOOs can increase the monoamine and BDNF levels in rodent depression models, which suggests its effect on depression.^26^, ^27^ In 2012, the CFDA approved MOOs as a prescribed medicine for minor or moderate depression. In our study, we have further demonstrated the effect of MOOs in alleviating depressive‐like behaviors in PSD rats, which may provide evidence for its use in the clinical treatment of PSD patients. Of note, the blood brain barrier is injured with high permeability after cerebral ischemic insult, even up to 1 month after stroke in rats,^38^ thus the orally administered MOOs can finally reach the brain to function.

Hippocampal inflammation has been extensively investigated in depression. Inhibition of hippocampal inflammation can alleviate LPS‐induced depressive behaviors in mice.^13^ Moreover, several clinical trials have illustrated that stroke patients with higher serum IL‐1β or IL‐18 levels at admission have a higher risk of developing PSD.^15^, ^16^ Here, we further demonstrated that inflammatory response was activated in the hippocampus of PSD rats and the microglial NLRP3 inflammasome activation was responsible for the excessive inflammatory response. However, it is interesting to note that the role of NLRP3 inflammasome in ischemic stroke is still controversial. In the study of Denes et al^39^, NLRP3^−/−^ and WT mice showed no difference on ischemic brain injury after MCAO, while absent in melanoma 2 (AIM2) and NLR family, CARD domain containing 4 (NLRC4) rather than NLRP3 inflammasome may contribute to brain injury.^40^, ^41^ This result is in contrast to a number of studies that have demonstrated the detrimental effect of NLRP3 inflammasome in ischemic stroke.^42^ Notwithstanding, the inflammation induced by NLRP3 inflammasome is a key factor in the pathogenesis of depression, which has been validated by both clinical and preclinical findings.^43^ PSD is defined as depression after a cerebrovascular incident and may share a common pathogenesis with depression. Thus, it is possible that NLRP3 inflammasome plays a role in the occurrence of PSD. Consistent with previous studies,^18^ our study confirmed that NLRP3 inflammasome is mainly activated in microglia in the hippocampus of PSD rats and MOOs can decrease NLRP3 inflammasome expression in PSD rats. Downregulation of hippocampal NLRP3 inflammasome abrogated depressive‐like behaviors and mitigated inflammatory response in the ischemic hippocampus of PSD rats. NLRP3 upregulation abrogates the effect of MOOs on depressive‐like behaviors and hippocampal inflammation response in PSD rats. Although our findings cannot fully elucidate the mechanism of PSD, they offer another insight into the correlation among hippocampal inflammatory response, microglial NLRP3 inflammasome activation and the occurrence of PSD. It is interesting to note that in the study of Wang et al,^20^ ameliorated NLRP3 activation can protect BBB integrity. However, the BBB is still broken after stroke compared with the intact rats. Thus, we think that this cannot affect MOOs across the BBB in PSD rats.

We further explored how MOOs regulate NLRP3 inflammasome and determined the NF‐κB signaling pathway. When suffering extracellular stimuli, IκB can be phosphorylated by kinase and subsequently dislocated from the NF‐κB dimer, followed by the translocation of NF‐κB into the cell nucleus.^44^ Nuclear NF‐κB can promote the transcription of NLRP3 initiating NLRP3 inflammasome activation.^45^ Additionally, the NF‐κB signaling pathway is activated in both depression^46^ and ischemic situations.^47^ In this study, MOOs suppressed the increase of phosphorylated IκB and NF‐κB p65 in both PSD rats and primary rat microglia, which could be involved in inhibited NLRP3 inflammasome activation. Notably, the regulation of NLRP3 inflammasome activation is a complex process and many other factors may also be involved, such as trans‐Golgi disassembly, lysosomal disruption, and K^+^ efflux.^17^ Thus, further investigation is needed to fully clarify the mechanism of MOOs on NLRP3 inflammasome activation inhibition.

One of the limitations of our studies is that the AAV‐NLRP3 we designed to regulate hippocampal NLRP3 inflammasome was not specifically targeted to microglia. Other cell types such as astrocytes and neurons may also be transfected with AAV‐NLRP3, resulting in decreased reliability of the results. However, consistent with other studies,^18^, ^48^ our work revealed that NLRP3 is mainly expressed on microglia and there is rare co‐localization of GFAP and NLRP3 in the hippocampus of PSD rats. Thus, it is possible that AAV‐NLRP3 mainly regulates NLRP3 expression in microglia rather than astrocytes. Future studies should be designed to specifically downregulate microglial NLRP3 inflammasome to verify our results. Additionally, we did not identify the specific molecular target of MOOs, because the major component of MOOs cannot be purified currently due to limitations in purification technology. Besides, the etiology of human PSD is very complex. Both social‐environmental risk factors (for instance, isolated residency, stressful life events, family relationships, and social support) as well as pathological characteristics of ischemic incident (for instance, ischemic site location, lesion size, the extent of edema and secondary leakage) increase the likelihood of developing PSD in stroke patients.^49^, ^50^ Thus, it is difficult to mimic clinical PSD in basic research, and currently several PSD animal models have been established, but all of the PSD models have their limitations. In our study, we used the “tMCAO + isolation + CUMS” PSD model, which is the most widely applied model in the basic research on PSD.^51^, ^52^, ^53^ However, environmental factors, such as family relationships and social support, cannot be stimulated in this model. Finally, we only used young male rats to study the effect of MOOs on microglia NLRP3 expression in our research. However, it is interesting to note that sex differences exist in microglial activation and neuroinflammation response after stroke.^54^ Sex differences in depressive‐like behavior may also be associated with the imbalance of microglia‐induced neuroinflammation in the hippocampus.^55^ Thus, it is possible that the effect of MOOs on microglia NLRP3 may be different in male and female rats. To fully elucidate this, both male and female PSD rats should be applied in further studies.

In conclusion, this study revealed the antidepressive effect and pharmacological mechanism of MOOs and emphasized NLRP3 inflammasome as the possible therapeutic target of PSD. We further illustrated that MOOs alleviate depressive‐like behaviors in PSD rats through the IκB/NF‐κB p65/NLRP3 inflammasome signaling pathway, which is an inflammatory pathway and may also offer insights for PSD treatment. Moreover, we proposed a correlation between stroke, hippocampal inflammation, and PSD. We further proposed the viewpoint that hippocampal inflammatory response activation followed by stroke could play a crucial role in the pathogenesis of PSD and that alleviating hippocampal inflammation through downregulating NLRP3 inflammasome is a potential novel target for PSD.

## 5 CONCLUSION

In this study, we confirmed the antidepressive effect of MOOs in PSD rat models. We also identified that MOOs can suppress microglial NLRP3 inflammasome activation to alleviate hippocampal inflammation. Moreover, we further illustrated that the IκB/NF‐κB p65 pathway can be inhibited by MOOs to downregulate NLRP3 inflammasome expression. The results shed light on NLRP3 inflammasome as a potential target for PSD treatment and highlight the involvement of hippocampal inflammation in PSD pathology.

## CONFLICT OF INTEREST

The authors declare that they have no conflict of interest.

## AUTHOR CONTRIBUTIONS

ZL and HX performed major experiments; GL performed the bioinformatic analysis; QP, JC analyzed data; RB and JL performed behavior tests; SC, YX, and HL reviewed the article; HJ and BH conceived the study and reviewed the article.

## Supporting information

Fig S1

## Data Availability

The datasets used in the current study are applicable from the corresponding author on reasonable request.
