# Supplementary figures and images for: Morinda officinalis oligosaccharides alleviate depressive‐like behaviors in post‐stroke rats via suppressing NLRP3 inflammasome to inhibit hippocampal inflammation
Source: CNS Neurosci Ther. 2021 Sep 24;27(12):1570–86. doi: 10.1111/cns.13732 (PMC8611777; doi:10.1111/cns.13732)

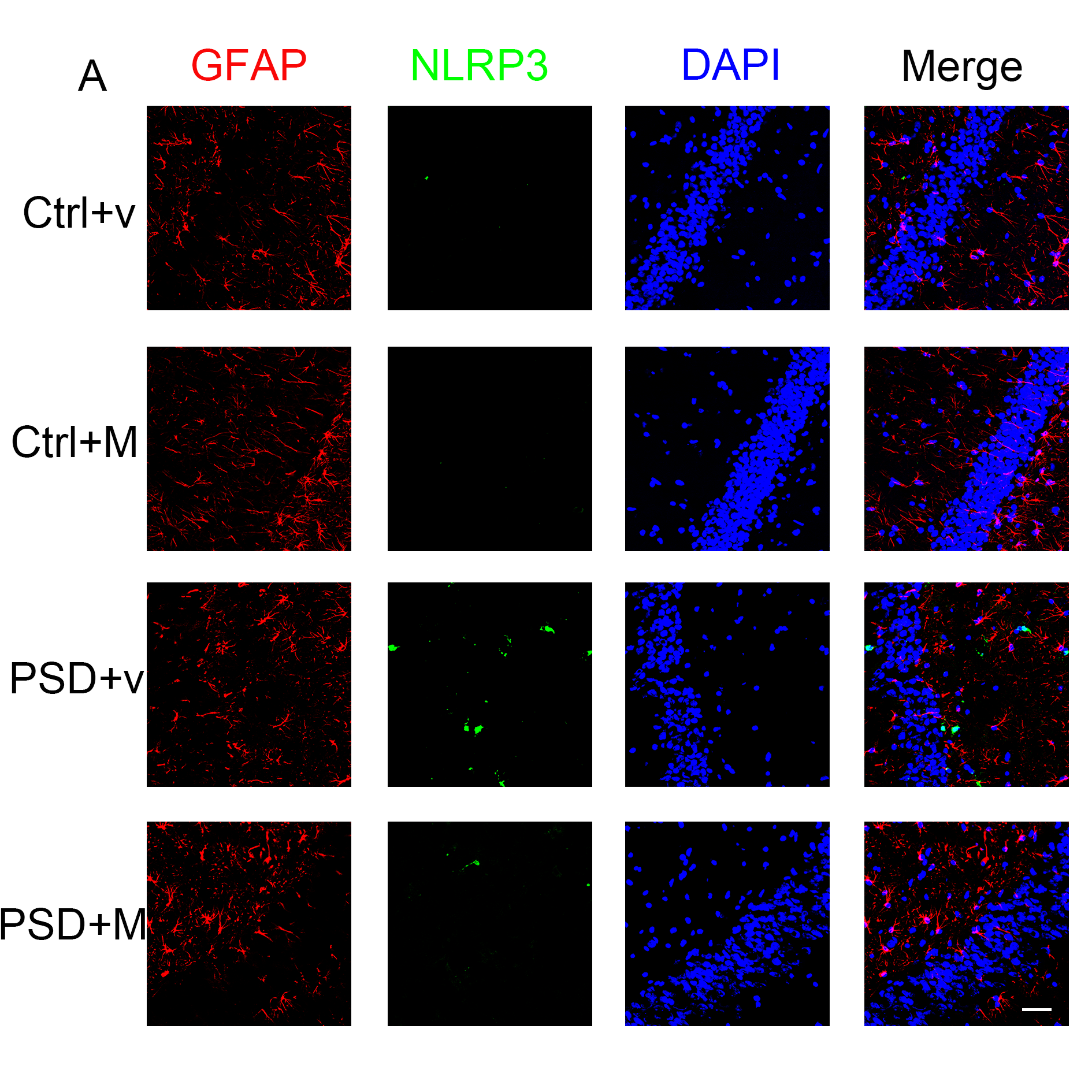

Supplement: Supplementary file 1 — Fig S1 [file CNS-27-1570-s001.tif]
